# Epidemiological and numerical simulation of rabies spreading from canines to various human populations in mainland China

**DOI:** 10.1371/journal.pntd.0009527

**Published:** 2021-07-14

**Authors:** Wen-gao Lu, Danni Ai, Hong Song, Yuan Xie, Shuqing Liu, Wuyang Zhu, Jian Yang

**Affiliations:** 1 School of Computer Science and Technology, Beijing Institute of Technology, Beijing, China; 2 School of Optics and Photonics, Beijing Institute of Technology, Beijing, China; 3 National Institute for Viral Disease Control and Prevention, Chinese Center for Disease Control and Prevention, Beijing, China; US Department of Agriculture, UNITED STATES

## Abstract

**Background:**

The mortality of humans due to rabies in China has been declining in recent years, but it is still a significant public health problem. According to the global framework, China strives to achieve the goal of eliminating human rabies before 2030.

**Methods:**

We reviewed the epidemiology of human deaths from rabies in mainland China from 2004 to 2018. We identified high risk regions, age and occupational groups, and used a continuous deterministic susceptibility-exposure-infection-recovery (SEIR) model with periodic transmission rate to explore seasonal rabies prevalence in different human populations. The SEIR model was used to simulate the data of human deaths from rabies reported by the Chinese Center for Disease Control and Prevention (China CDC). We calculated the relative transmission intensity of rabies from canines to different human groups, and they provided a reliable epidemiological basis for further control and prevention of human rabies.

**Results:**

Results showed that human deaths from rabies exhibited regional differences and seasonal characteristics in mainland China. The annual human death from rabies in different regions, age groups and occupational groups decreased steadily across time. Nevertheless, the decreasing rates and the calculated R_0_s of canines of various human groups were different. The transmission intensity of rabies from canines to human populations was the highest in the central regions of China, in people over 45 years old, and in farmers.

**Conclusions:**

Although the annual cases of human deaths from rabies have decreased steadily since 2007, the proportion of human deaths from rabies varies with region, age, gender, and occupation. Further enhancement of public awareness and immunization status in high-risk population groups and blocking the transmission routes of rabies from canines to humans are necessary. The concept of One Health should be abided and human, animal, and environmental health should be considered simultaneously to achieve the goal of eradicating human rabies before 2030.

## Author summary

Animal and human rabies were still significant public health problems in China. In the past 15 years, although the annual human deaths from rabies have decreased steadily, the proportion change in human deaths from rabies varied with diverse population groups. This condition reflected the different recognition and attention to rabies of various populations. We applied a continuous deterministic SEIR model to study the spread of rabies among canines and from canines to different human groups. Numerical simulation results showed that the proportion of human deaths from rabies in central regions, the populations over 45 years old and the farmers in China increase year by year, and transmission intensity of rabies from canines to the above groups are higher than the nationwide level. Finally, our research indicated that to achieve the ultimate goal of eliminating human rabies by 2030, the immunization status of high-risk human groups and mammals in corresponding areas should be enhanced, and the cooperation with various departments related to human, animal and the environment should be enhanced in line with the concept of One Health.

## Introduction

Rabies is an acute and fatal zoonosis caused by genus *Lyssavirus* viruses[1], which can infect humans and mammals, including livestock and pets. When rabies virus enters the human body, it spreads rapidly along the neural pathway and spreads to other organs, intrudes on various tissues and causes morbidity. Without timely and effective immunization, humans infected with rabies virus experience an incubation period of months to years before symptoms appear[2]. Once rabies virus reaches the central nervous system and causes symptoms, the course of disease usually does not exceed 10 days, and the mortality rate is as high as 100%. Mammalian rabies can be prevented by vaccination, and treatment of humans, namely, post exposure prevention (PEP) after exposure can prevent human rabies effectively.

Human rabies is widely distributed around the world, and tens of thousands of people lose their lives every year[3]. After years of active immunization, human rabies has almost disappeared in Europe and North America[4]. However, in numerous developing and the least developed countries and regions in Asia and Africa, rabies is still a neglected and untreatable public health problem. In these areas, canines are the main hosts of rabies virus. The transmission of rabies virus from infected canines to humans through bites or scratches has still been the main cause of human deaths from rabies worldwide. Statistical analysis indicated that animal rabies[5,6] and human rabies[7] exhibited seasonal periodicity, and the morbidity in summer and autumn was significantly higher than that in winter and spring. Hutter et al.[8] concluded that the changing weather and the El Nino Southern Oscillation could affect cattle rabies outbreaks and mortality in Costa Rica. Yao et al.[9] revealed that the occurrences of human rabies in mainland China were related to natural environments and sociological factors, including temperature and regional economy, through spatial correlation analysis. According to the World Health Organization (WHO), the annual deaths of human rabies in mainland China is only second to India[10], thereby confirming that human rabies is still a major public health problem in mainland China[11].

Mathematical modeling has become a significant tool to analyze the epidemiological characteristics of infectious diseases. This approach can provide effective guidance for the prevention and control of infectious diseases[12]. Aiming to study the prevalence of rabies in humans and animals, researchers have established dynamic models including SEIR groups to explain the transmission dynamics of rabies among mammals, including canines and bats, and between mammals and humans. Anderson et al.[13] first proposed a deterministic model including susceptible, exposed, and recovered groups to comprehend the epidemiologic features of fox rabies in Europe. Childs et al.[14] predicted the dynamic change in raccoon rabies in the United States. Dimitrov et al.[15,16] extended the SEIR model to an individual model of rabies virus immune response of bats to investigate the ecological characteristics of bats in different habitats after infection. Georgea et al.[17] described the seasonal characteristics of bat rabies under the influence of host and virus ecology. Clayton et al.[18] studied the population dynamics of raccoons and discussed the influence of vaccination and seasonal birth pulse on raccoon rabies. Hampson et al.[19] observed the canine rabies in sub Saharan Africa and found that rabies prevention measures are affected by disease prevalence. They showed that a simple model with intervention responses could capture observed disease periodicity and host dynamics.

These studies using the SEIR model are limited to the rabies characteristics of one single species. In recent years, researchers have focused on the transmission effects between different species or different groups in one species. Zinsstag et al.[20,21] analyzed the transmission dynamics and the economics of canine and human rabies in African cities. They pointed out that the combination of canine vaccination campaign and human vaccination plan was more cost-effective. Zhang et al.[22] developed a deterministic model to explore the spread of rabies from canines to humans in China. They divided the population of humans and canines into four subgroups (S, E, I and R) and found that reducing the incidence rate of rabies and increasing the immunity rate of canines were the most effective to control human rabies in China through numerical simulation and sensitivity analysis by using the annual human deaths of rabies from 1996 to 2010 in China. They also found that killing of canines can be replaced by extensive immunization to hinder the spread of rabies. Hou et al.[23] divided the canine populations into domestic and stray canines and studied the spread of rabies from different canine groups to humans, applying the SEIR model. They considered that the domestic canine vaccination rate, the recruitment rate of domestic canines, and the quantity of stray canines played important roles in the spread of rabies through numerical simulation using the annual human death of rabies in Guangdong Province, China. Huang et al.[24] built a multihost transmission dynamic model among canines, Chinese ferret badger, and humans and predicted the development of human and livestock rabies.

The outbreak of rabies was characterized by periodicity and spatial transmission. Thus, Zhang et al.[25,26] extended the SEIR model with periodic transmission rate and established a reaction diffusion dynamic model between canines and humans to study the seasonal epidemic and the propagation speed of rabies. They showed that the movement of canines greatly influenced the propagation wave speed. Chen et al.[27] built a multipatch SEIR model to study the effect of dynamic migration of canines and humans between different pairs of provinces in China and the sensitivity of migration rate of canines to the basic reproduction numbers. Tian et al.[28] combined the SEIR model and phylogeographic analyses to precisely analyze the transmission of human rabies in rural areas of Yunnan Province, China.

However, the previous reports did not focus on human rabies of different population subgroups, including different age, gender, and occupation groups, of one country or region. The comparison of rabies transmitted from mammals to different human groups in one region has not been reported. We initially described the latest epidemiology of human rabies in mainland China and identified high-risk regions, age groups, and occupational groups to assist human rabies prevention. To understand the spread of rabies from canines to different human groups in China and explore effective control and prevention measures, considering that people usually wore thin clothes, had more outdoor activities, and canines were more manic in summer[25], we established a continuous deterministic and periodic SEIR model to describe the spread of rabies between canines and different human groups. We also compared and evaluated the basic reproduction numbers of canines, and calculated the relative transmission intensity of rabies from canines to different population groups to identify people with higher risk of rabies.

## Methods

### Ethics statement

According to the National Health Commission of the People’s Republic of China, data from human rabies cases are collected to monitor notifiable infectious diseases without the assessment of the institutional review committee.

### Data collection

Human rabies was a significant public health problem in China, and it was listed as a Class B notifiable infectious disease. After 2003, the Chinese government constructed the national notifiable disease reporting system and required clinicians to report the personal information of patients within 24 hours after confirmation, including gender, date of birth, date of onset, and place of residence, to China CDC online using standardized forms. The dataset of Chinese human deaths from rabies from 2004 to 2018, including number of deaths per month by each province, age and gender groups, and occupations were outputted from the national notifiable disease reporting system and provided by National Institute for Viral Disease Control and Prevention in China CDC. The total populations of each group were referred to the China Statistical Yearbook. They were provided by the National Bureau of Statistics of China every year[29].

### Descriptive statistics

We calculated the frequencies, means and standard deviations of normal distribution, time series trend of numbers of human deaths from rabies of different regions, age groups, and occupational groups from 2014 to 2018, and did specific hypothesis tests introduced next to estimate the proportions of annual human deaths from rabies of different subgroups. The Chi-square test was used to monitor whether the proportion of human deaths from rabies every year in different subgroups exhibited statistically different. The Mann Kendall trend test was used to examine whether there was a significant downward or upward trend in the proportion of human deaths from deaths of various subgroups. Significance tests were two-tailed, and p<0.05 was considered statistically significant. The above steps helped to determine the classification of subgroups in numerical analysis. We examined human deaths due to rabies based on four demographic categories: region, age, gender, and occupation. For simplicity, we subjectively grouped each category based on post-hoc examinations of the proportion of human deaths from rabies. In particular, we divided the total population in mainland China into five regions, and the age was split into under 45 years old group and over 45 years old group. The occupations were grouped into four categories: farmers, students, children, workers and other occupations. In particular, the children group includes scattered children and children in kindergartens, mainly under 6 years old before primary school; the students group includes primary school students, middle school students, college and university students; the workers group includes various unskilled workers; and the other occupations include teachers, doctors, catering practitioners, waiters, business service providers, medical personnel, seafarers and drivers, cadre staff, herdsmen, fishermen, and retirees.

The SEIR model was applied to simulate the monthly time series of human deaths from rabies of various human groups from 2014 to 2018 to study the spread of rabies from canines to various human groups. The descriptive statistics and numerical simulation using the SEIR model were performed using Python 3.7. The Relative Error was applied to obtain the optimum fitting parameters.

### Mathematical model and sensitivity analysis

**Fig 1:**
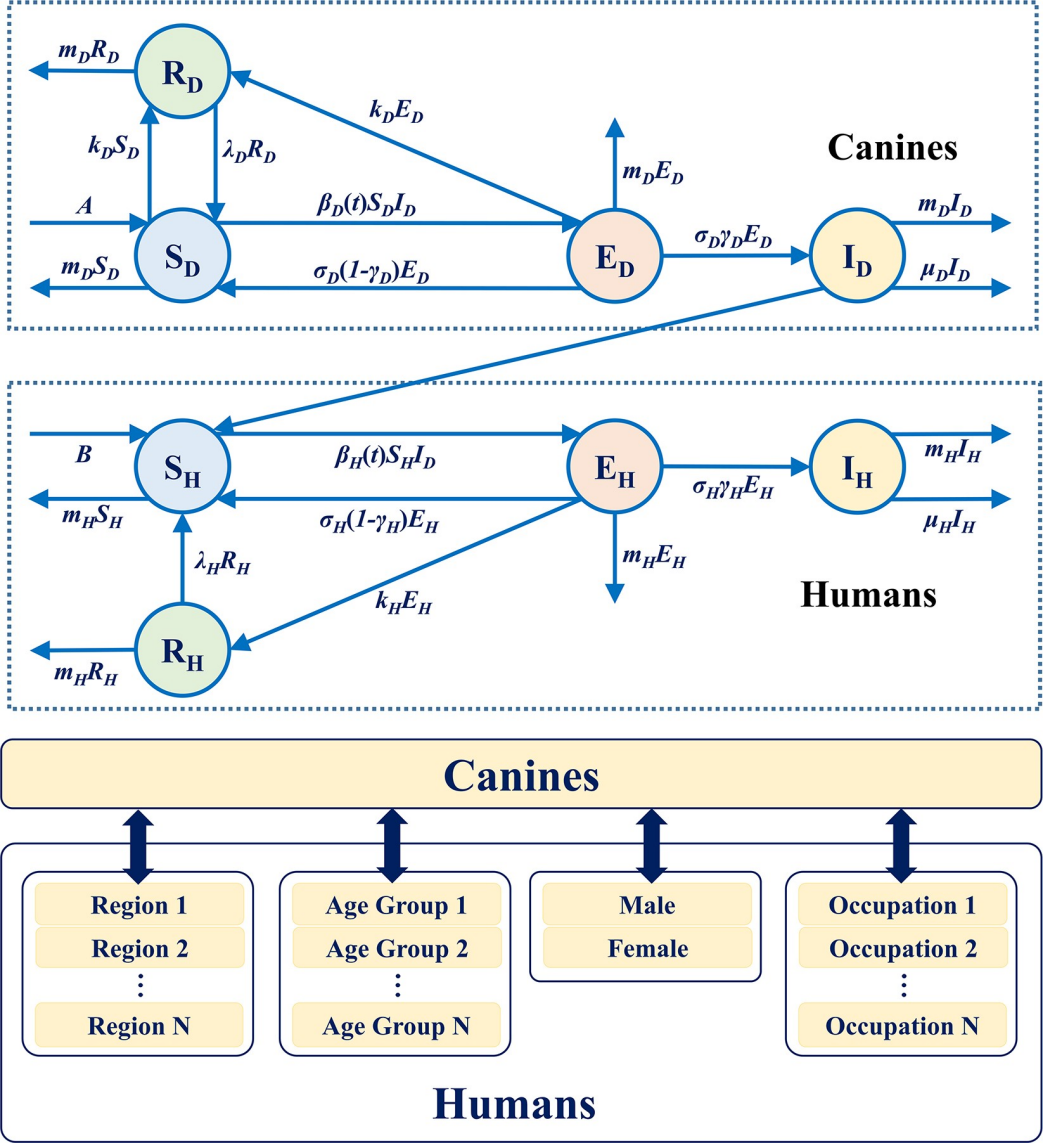
Transmission model of rabies among canines and from canines to various human groups.


We extended the continuous deterministic SEIR model of canine rabies to analyze the seasonal periodic mode of rabies of different human groups in China. We could perform numerical simulation by using the overall mortality and the mortality of different human groups separately, to study the transmission of rabies in canines and different human groups, considering that almost no cross transmission of rabies was found among humans. The canine groups and different human groups were divided into four subclasses, namely, susceptible, exposed, infected, and recovered groups. Then, the SEIR model was built on the basis of eight subclasses, and the related parameters of various groups are explained in Fig 1. The mathematical model, which consists of eight ordinary differential equations, was built, as shown in Eq (1).

$$\left\{ \begin{array}{l} \frac{dS_{D}}{dt}=A+\lambda_{D}R_{D}+\sigma_{D}(1-\gamma_{D})E_{D}-\beta_{D}(t)S_{D}I_{D}-(m_{D}+k_{D})S_{D}\\ \frac{dE_{D}}{dt}=\beta_{D}(t)S_{D}I_{D}-(m_{D}+\sigma_{D}+k_{D})E_{D}\\ \frac{dI_{D}}{dt}=\sigma_{D}\gamma_{D}E_{D}-(m_{D}+\mu_{D})I_{D}\\ \frac{dR_{D}}{dt}=k_{D}(S_{D}+E_{D})-(m_{D}+\lambda_{D})R_{D}\\ \frac{dS_{H}}{dt}=B+\lambda_{H}R_{H}+\sigma_{H}(1-\gamma_{H})E_{H}-\beta_{H}(t)S_{H}I_{D}-m_{H}S_{H}\\ \frac{dE_{H}}{dt}=\beta_{H}(t)S_{H}I_{H}-(m_{H}+\sigma_{H}+k_{H})E_{H}\\ \frac{dI_{H}}{dt}=\sigma_{H}\gamma_{H}E_{H}-(m_{H}+\mu_{H})I_{H}\\ \frac{dR_{H}}{dt}=k_{H}E_{H}-(m_{H}+\lambda_{H})R_{H} \end{array}\right.$$ (1)

where

$$\beta_{D,H}=a_{D,H}[1+b_{D,H}sin(\pi t/6+5.5)]$$ (2)

which was proposed by Schenzle, D.[30] to describe the periodic transmission rates among canines and from canines to humans.

In Eq (1), the subscripts D and H represent canines and humans, respectively. For canines, A describes the birth population rate, λ_D_ denotes the loss rate of vaccination immunity of canines, γ_D_ is the risk factor of clinical outcome of exposed canines, σ_D_ is the time duration of infected canines that remain infectious, and its reciprocal represents the incubation period of canines. Thus, σ_D_γ_D_E and σ_D_(1-γ_D_)E represent the exposed canines that develop clinical rabies or do not develop clinical rabies and return to the susceptible class, respectively. m_D_ is the natural mortality rate of canines, β_D_ is the transmission rate among canines, in which a_D_ and b_D_ are baseline and magnitude of β_D_ for data analysis within a month period, respectively. k_D_ is the vaccination rate of canines, μ_D_ is the death rate of canines due to rabies. For humans, B describes the birth population rate, λ_H_ denotes the loss rate of vaccination immunity of humans, γ_H_ is the risk factor of clinical outcome of exposed humans, σ_H_ is the time duration of the infected humans that remain infectious, and its reciprocal represents the incubation period of humans. Thus, σ_H_γ_H_E and σ_H_(1-γ_H_)E represent the exposed humans that develop clinical rabies or do not develop clinical rabies and return to the susceptible class, respectively. m_H_ is the natural mortality rate of humans, β_H_ is the transmission rate from canines to humans, in which a_H_ and b_H_ are the baseline and magnitude of β_H_ for data analysis within a month period, respectively. k_H_ is the vaccination rate of humans, μ_H_ is the death rate of humans due to rabies. The parameters and values in unit months^-1^ are listed in Tables 1 and S1. The values of most parameters were estimated by referring to the previous reports, as listed in Table 1. Part of the parameters, including a_D_, b_D_, a_H_, and b_H_, were obtained through numerical simulations. The birth rate of canines was estimated by referring to an internal file provided by several monitoring sites of China CDC. This parameter was set as constant in the SEIR model for studying the transmission of rabies from canines to human groups including the nationwide, different age, gender and occupation groups, and was set according to the proportion of population nationwide for various regions of China, as listed in S1 Table.

**Table 1:** Descriptions and values of parameters in model (1).

Parameters	Value	Unit	Description	Source
**A**	2.34×10^5^	month^-1^	Birth population rate of canines	Assumption
**λ**_**D**_	0.1666	month^-1^	Loss rate of vaccination immunity of canines	Assumption
**γ**_**D**_	0.49	month^-1^	Clinical outcome rate of exposed canines	[20]
**σ**_**D**_	0.95	month^-1^	Reciprocal of the canine incubation period	[20]
**m**_**D**_	0.0064	month^-1^	Natural mortality rate of canines	[25]
**a**_**D**_	9.95×10^−8^	-	The baseline transmission rate among canines	Fitting
**b**_**D**_	0.42	-	The magnitude of forcing	Fitting
**k**_**D**_	0.09	month^-1^	Vaccination rate of canines	[32]
**μ**_**D**_	1	month^-1^	Disease related death rate of canines	[33]
**B**	1.34×10^6^	month^-1^	Birth population rate of humans	[31]
**λ**_**H**_	0.1666	month^-1^	Loss rate of vaccination immunity of humans	Assumption
**γ**_**H**_	0.5	month^-1^	Clinical outcome rate of exposed humans	[33]
**σ**_**H**_	0.5	month^-1^	Reciprocal of the human incubation period	[33]
**m**_**H**_	0.000642	month^-1^	Natural mortality rate of humans	[31]
**a**_**H**_	2.41×10^−11^	-	The baseline transmission rate of canines to humans	Fitting
**b**_**H**_	0.33	-	The magnitude of forcing of canines to humans	Fitting
**k**_**H**_	0.54	month^-1^	Vaccination rate of humans	[32]
**μ**_**H**_	1	month^-1^	Disease related death rate of humans	[33]
**S**_**D**_	3.30×10^7^	-	Initial value of susceptible canines	[32]
**E**_**D**_	2.20×10^4^	-	Initial value of exposed canines	[32]
**I**_**D**_	1.10×10^4^	-	Initial value of infectious canines	[32]
**R**_**D**_	3.30×10^6^	-	Initial value of recovered canines after vaccination	[32]
**S**_**H**_	1.30×10^9^	-	Initial value of susceptible humans	[31]
**E**_**H**_	286	-	Initial value of exposed humans	China CDC
**I**_**H**_	143	-	Initial value of symptomatic humans	China CDC
**R**_**H**_	6.00×10^7^	-	Initial value of recovered humans after vaccination	[32]

The basic reproduction number (R_0_) of canine rabies can be calculated by the formula provided by the previous article[25]. In this case, R_0_ is expressed by formula (3).

$$R_{0}=\frac{\sigma_{D}\bar{\beta }S^{0}\gamma_{D}}{\left(m_{D}+\sigma_{D}+k_{D})(m_{D}+\mu_{D}\right)}$$ (3)

where

$$\left\{ \begin{array}{l} S^{0}=\frac{(m_{D}+\lambda_{D})A}{m_{D}(m_{D}+\lambda_{D}+k_{D})}\\ \bar{\beta }=\frac{1}{12}\int_{0}^{12}\beta (t)dt \end{array}\right.$$ (4)

R_0_ is an important measure of the number of healthy individuals that can be infected by infectious individuals during the infection period. If R_0_ is greater than 1, then an infectious canine will spread rabies to more than one healthy canine on the average, thereby leading to the long existence of rabies. On the contrary, if R_0_ is less than 1, then the rabies will gradually disappear because of the insufficient transmission rate. Considering different levels of prevention and control measures can affect the value of R_0_ for one single infectious disease. Therefore, the length of period to control the disease can be determined. Previous studies have shown that the transmission of rabies can be effectively controlled by immunization of 70% of the canines[22].

Sensitivity analysis will be conducted by simulating the influence of changing the birth rate or the immunization rate of canines on human deaths from rabies and the basic reproduction numbers, so as to explore an effective way to control human rabies.

## Results

### Descriptive statistics results

We conducted proportional statistics and mean tests on the time series of human deaths from rabies of the nationwide and various regions, gender, age, and occupation groups in mainland China. The Chi-square test was used to monitor whether the proportion of human deaths from rabies every year in different subgroups exhibited statistically different. The Mann Kendall trend test was used to examine whether there was a significant downward or upward trend in the proportion of human deaths from deaths of various subgroups to explore the risk of rabies in different population groups.

### Overall and seasonal epidemic trend

**Fig 2:**
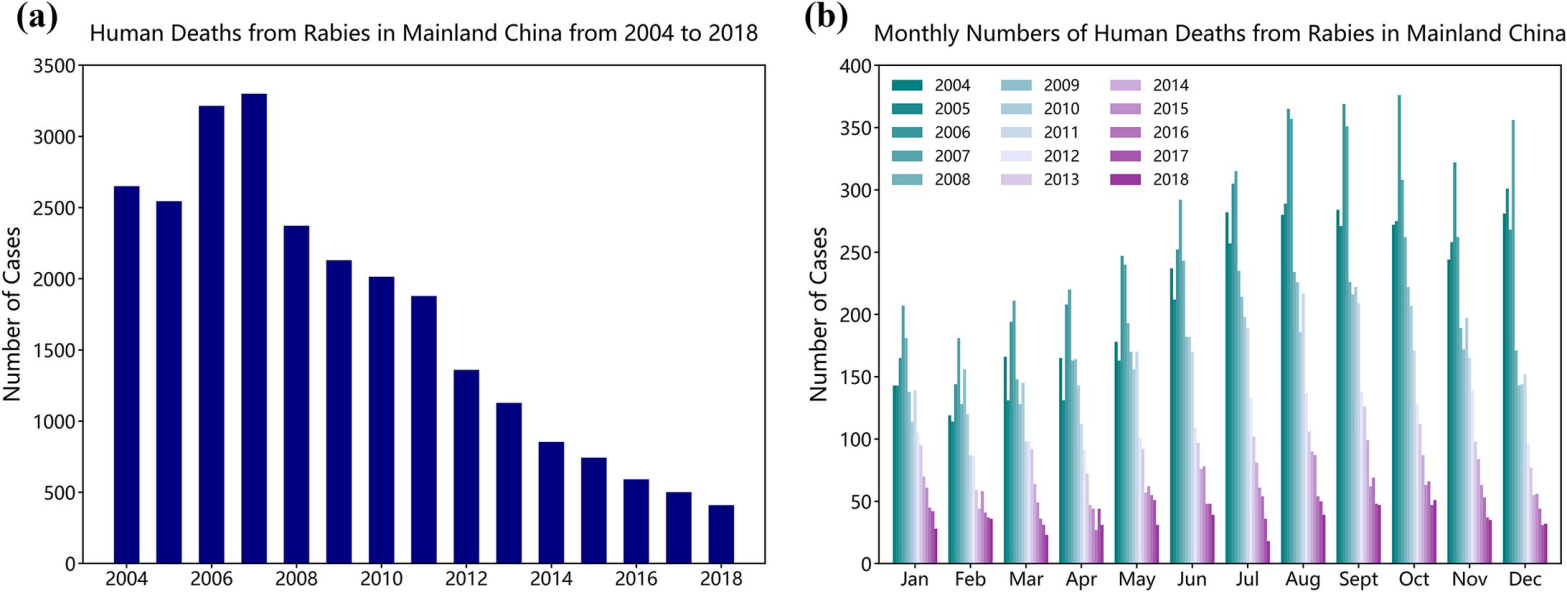
Time series of human deaths from rabies in mainland China. (a) Annual human deaths from rabies in mainland China ranging from 2014 to 2018. (b) Seasonal pattern of human deaths from rabies in mainland China.

From 2004 to 2018, 25699 cases of human deaths from rabies were reported in mainland China. As shown in Fig 2A, the annual human deaths from rabies reached a peak of 3300 in 2007. Since then, due to the continuous strengthening of prevention and control policies, the annual human deaths from rabies have decreased year by year. In 2018, the number of cases has dropped to 410, less than one eighth of that in 2007. Meanwhile, the mortality of human deaths from rabies in China exhibited significant seasonality characteristics. As shown in Fig 2B, the numbers of human deaths from rabies in summer and autumn were higher, with the average proportion in September reaching 10.61% (95% CI = (8.78%, 12.44%)), and the average proportion in February being the lowest, 5.99% (3.30%, 8.67%). The Chi-square test indicated no statistical difference between different years (p > 0.05).

### Regional distribution

**Fig 3:**
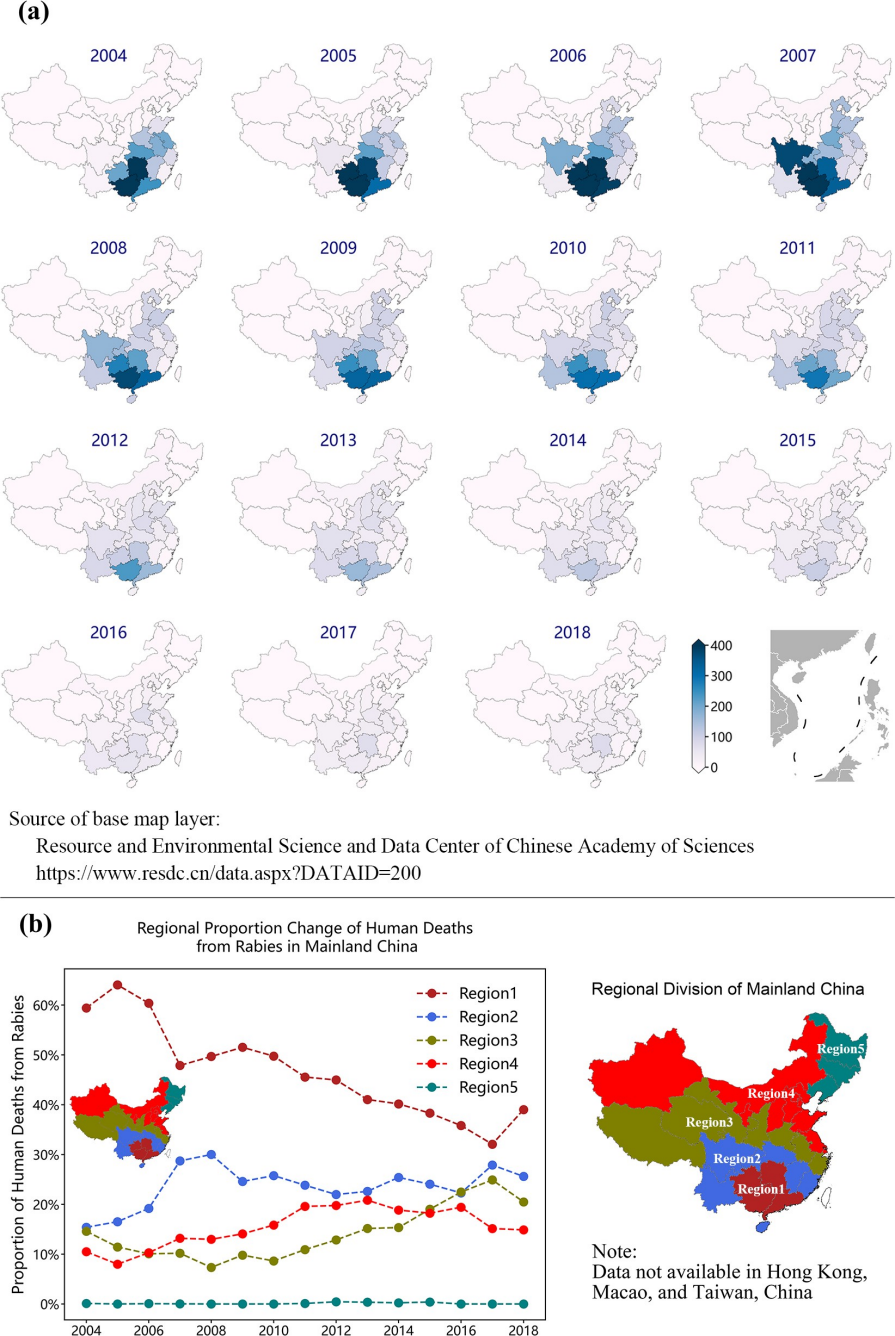
The regional distribution of human deaths from rabies in mainland China. (a) The annual human deaths from rabies in different states and territories from 2004 to 2018 in mainland China. (b) The proportion change of the human deaths from rabies in various regions from 2004 to 2018 in mainland China. Source of the base map layer: Resource and environmental science and data center of Chinese Academy of Sciences, https://www.resdc.cn/data.aspx?DATAID=200.

The regional distribution of human deaths from rabies in mainland China is illustrated in Fig 3A. From 2004 to 2018, the annual human deaths from rabies in various provinces generally experienced a process of first growth followed by steady decline after 2007. Meanwhile, the human deaths from rabies exhibited distinct regional differences, and the southwest of China was the highest risk area. The top four states and territories of annual mortality are Guangxi Zhuang Autonomous Region, which accounted for 14.64% (7.26%, 22.01%), followed by Hunan Province, 11.26% (3.38%, 19.15%), Guizhou Province, 10.47% (2.31%, 18.62%), and Guangdong Province, 10.27% (3.68%, 16.87%). We defined these states and territories as region I (high risk area) in the following mathematical modeling analysis. However, the proportion of human deaths from rabies reported in region I has evidently declined from approximately 60% in 2004 to lower than 40% in 2018, as shown in Fig 3B. This finding indicated that canine and human rabies may have been spread from south China to the north. On the basis of these considerations, we divided mainland China into five nonoverlapping regions in the mathematical modeling analysis. The set of states and territories bordering region I are defined as region II, as shown in Fig 3B, padding using blue color. The set of states and territories bordering region II (excluding four provinces of region I) is defined as region III, and the others are defined as regions IV and V. Mann-Kendall trend test explained that the proportion of annual human deaths from rabies of region I had a significant downward trend (p < 0.01), whereas the proportions of those in regions III and IV increased (p < 0.05). This finding demonstrated the spread of canine and human rabies in mainland China from the south to the north.

### Demographic features

**Fig 4:**
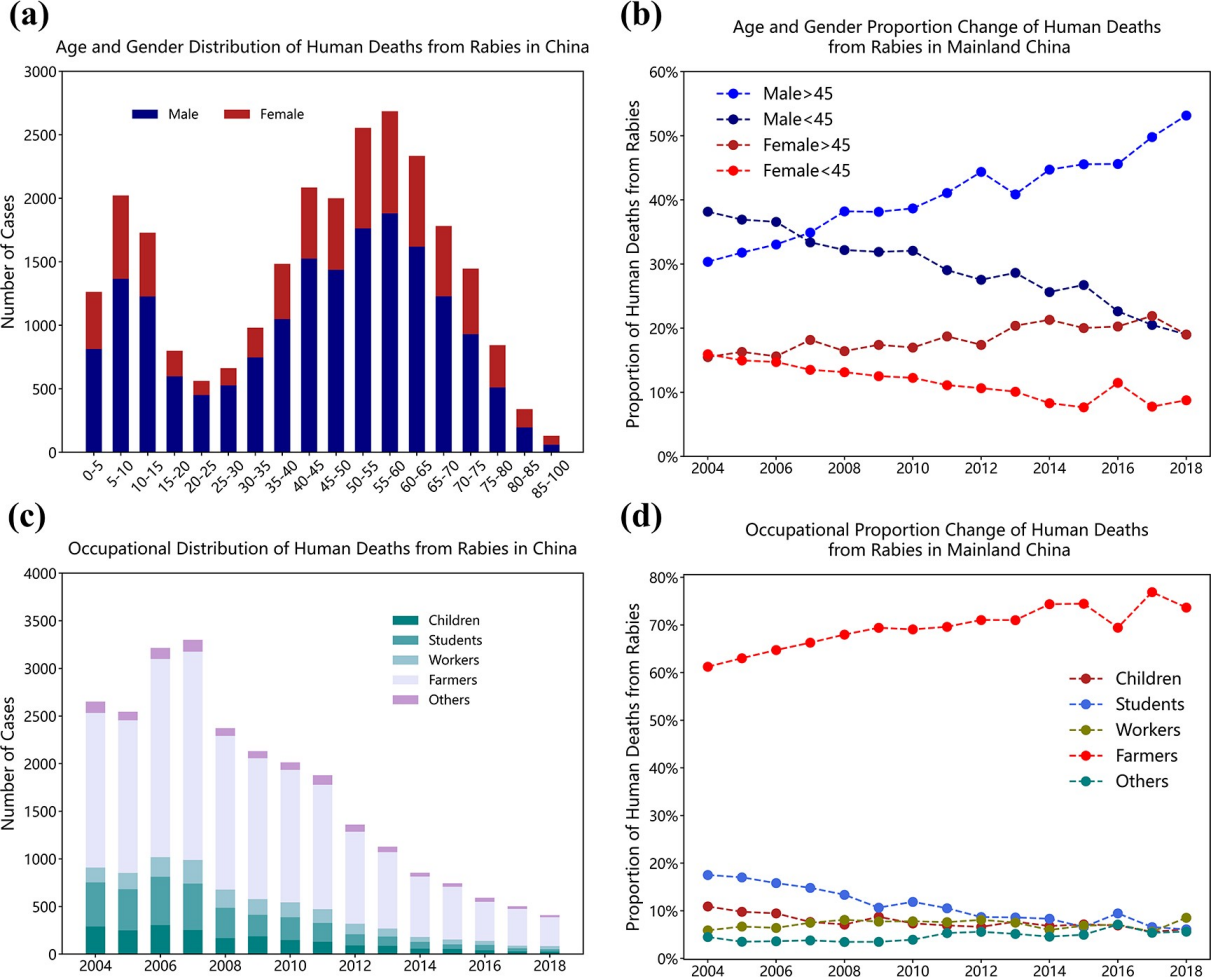
The demographic characteristics of human deaths from rabies in mainland China. (a) Age group and gender distribution of annual human deaths from rabies of from 2004 to 2018 in mainland China. (b) The proportion change of male and female human deaths from rabies above and below 45 years old. (c) Occupational group distribution of annual human deaths from rabies from 2004 to 2018. (d) The proportion change of human deaths from rabies of different occupational groups.

Human deaths from rabies in mainland China vary with gender, age, and occupation. Fig 4A illustrates the age group distribution of annual human deaths from rabies of men and women from 2004 to 2018. Statistics suggested a significant difference in the number of human deaths from rabies between different age groups (p < 0.001), among which men and women ranging from 55 to 60 years old accounted for the highest proportion. The mortality ratio of men to women in all age groups was 2.37 (0.79, 3.95), and no statistical difference was found between different years (p > 0.05). However, the proportions of human deaths from rabies in different age groups changed. The proportion of annual mortality of the population under 45 years old generally decreased, (for males, 0 to 20 years old, and 30 to 40 years old groups; for females, 0 to 25 years old, and 30 to 45 years old groups, p < 0.05). However, the proportion of annual mortality in the population over 45 years old was opposite (for males, 45 to 50 years old, and over 60 years old groups; for females, 60 to 75 years old, and 80 to 85 years old groups, p < 0.05) according to the Mann-Kendall trend test, as illustrated in Fig 4B. The detailed test results are listed in S2 Table. Therefore, we believe that different age populations have different risks for human rabies. In accordance with the proportion changes in the prevalence of human deaths from rabies in different age groups, we divided men and women in mainland China into two groups when using the SEIR model, as follows: under 45 year old group and above 45 year old group.

In terms of occupation, the annual mortalities of different occupational groups are shown in Fig 4C. Statistics suggested a significant difference in the number of human deaths from rabies between different occupational groups, among which the farmers accounted for the largest proportion, 69.48% (69.85%, 78.10%), followed by students 11.06% (3.53%, 18.59%), children 7.65% (4.79%, 10.51%), workers 7.15% (5.40%, 8.91%), and other professional groups accounted for only 4.66% (2.59%, 6.73%) in total. However, the proportions of annual mortality of different occupational groups have also changed. According to the Mann-Kendall trend test, the proportion of farmers increased significantly (p < 0.01), and the result was the opposite for students and children (p < 0.01). Meanwhile, the proportion of workers and the three subgroups of workers exhibited no significant trend (p > 0.05), as illustrated in Fig 4D. The proportion of the other professional groups exhibited no significant trend (p > 0.05). As the monthly human deaths from rabies of each profession in other occupational groups was very rare (SEIR model cannot be used for numerical simulation), and the total number of annual human deaths from rabies of other occupational groups (including unknown occupational groups) was very small (only 23 in total in 2018), we believe human rabies has been effectively controlled in the other professional groups. Therefore, in the following mathematical modeling analysis, we divided the population in mainland China into four major occupation groups, including farmers, students, children, and workers, whereas the other professional groups were not included in the numerical simulations.

### Mathematical model results

In this section, the SEIR model was applied to simulate the monthly time series of human deaths from rabies of various human groups from 2014 to 2018 to study the spread of rabies from canines to various human groups, and the basic reproduction numbers were calculated for the representation of the transmission intensity of rabies from canines to different human populations. Sensitivity analysis was conducted to explore the effective measures to control the spread of rabies in canines and from canines to humans.

### Numerical simulation and the basic reproduction numbers

We analyzed the transmission among canines and between canines and various human populations by using a continuous deterministic SEIR model. Demographic information, including annual human birth rate, mortality rate, age group distribution and regional distribution of population were consulted from China Statistical Yearbooks[31]. All the parameters of SEIR model based on the nationwide scope are listed in Table 1, and all the parameters based on other human groups are listed in S1 Table. In the research of the populations under 45 years old, m_H_ represents the rate of exceeding 45 years old. In the research of the populations over 45 years old, B represents the rate of exceeding 45 years old. For different occupational groups, B is estimated according to the number of new entrants each year, and m_H_ represents the graduation ratio (for students), the rate of exceeding childhood age (for children), and death rate (for farmers). The initial values of these parameters were set by referring to the previous reports because we could not accurately obtain the exact values of the SEIR canine populations, as listed in Table 1. The initial values of the number of workers and farmers were estimated in accordance with the employment situation in 2004 by referring to the China Statistical Yearbook[31].

**Fig 5:**
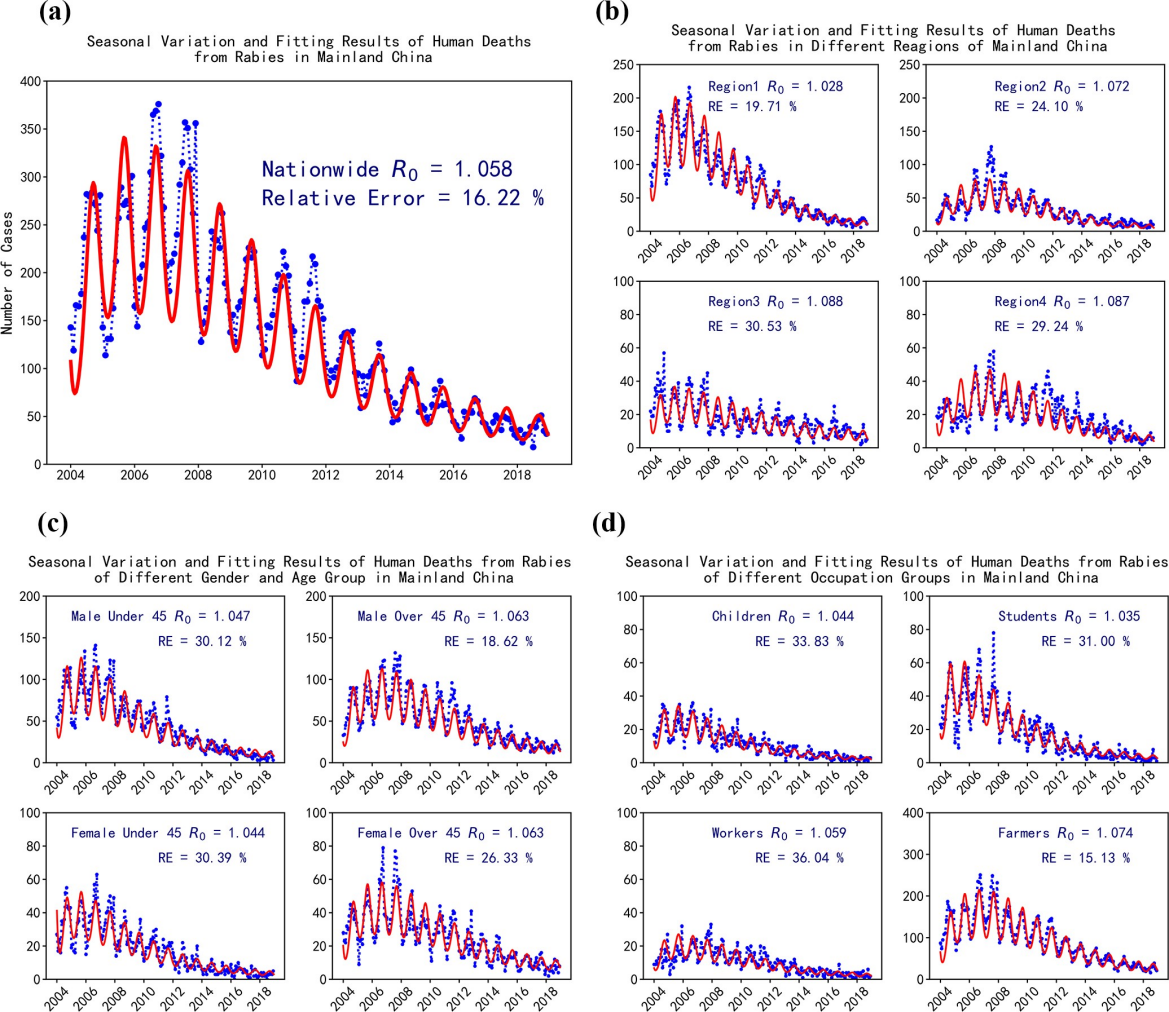
Numerical simulation based on human deaths from rabies of different populations. (a) Numerical simulation based on nationwide data of human deaths from rabies. (b) Numerical simulation based on human deaths from rabies of various regions in mainland China. (c) Numerical simulation based on human deaths from rabies of various age and gender groups. (d) Numerical simulation based on human deaths from rabies of various occupational groups.

Numerical simulation results based on the human deaths from rabies in mainland China are shown in Fig 5A, and those of various human populations, including different regions, and age and gender and occupational groups, are shown in Fig 5B, 5C and 5D. In all the figures, the blue dotted lines represent the number of human deaths from rabies reported in each month from 2004 to 2018, and the red curved lines are the numerical simulation results, which were in agreement with the reported data (Relative Error = 16.22% nationwide, as shown in Fig 5A). The figures show that, less human deaths from rabies led to violent fluctuation. Thus, the simulation results can still reflect the stable downward trend and the seasonal mode. The nationwide mortality related to rabies reached a peak in 2007, and then it decreased with annual oscillation due to the continuous strengthening of prevention and control forces. However, the years in which the decline began were different for the different population groups. The number of human deaths from rabies in region I reached a peak in 2006, and those in other groups reached their peaks in 2007.

**Fig 6:**
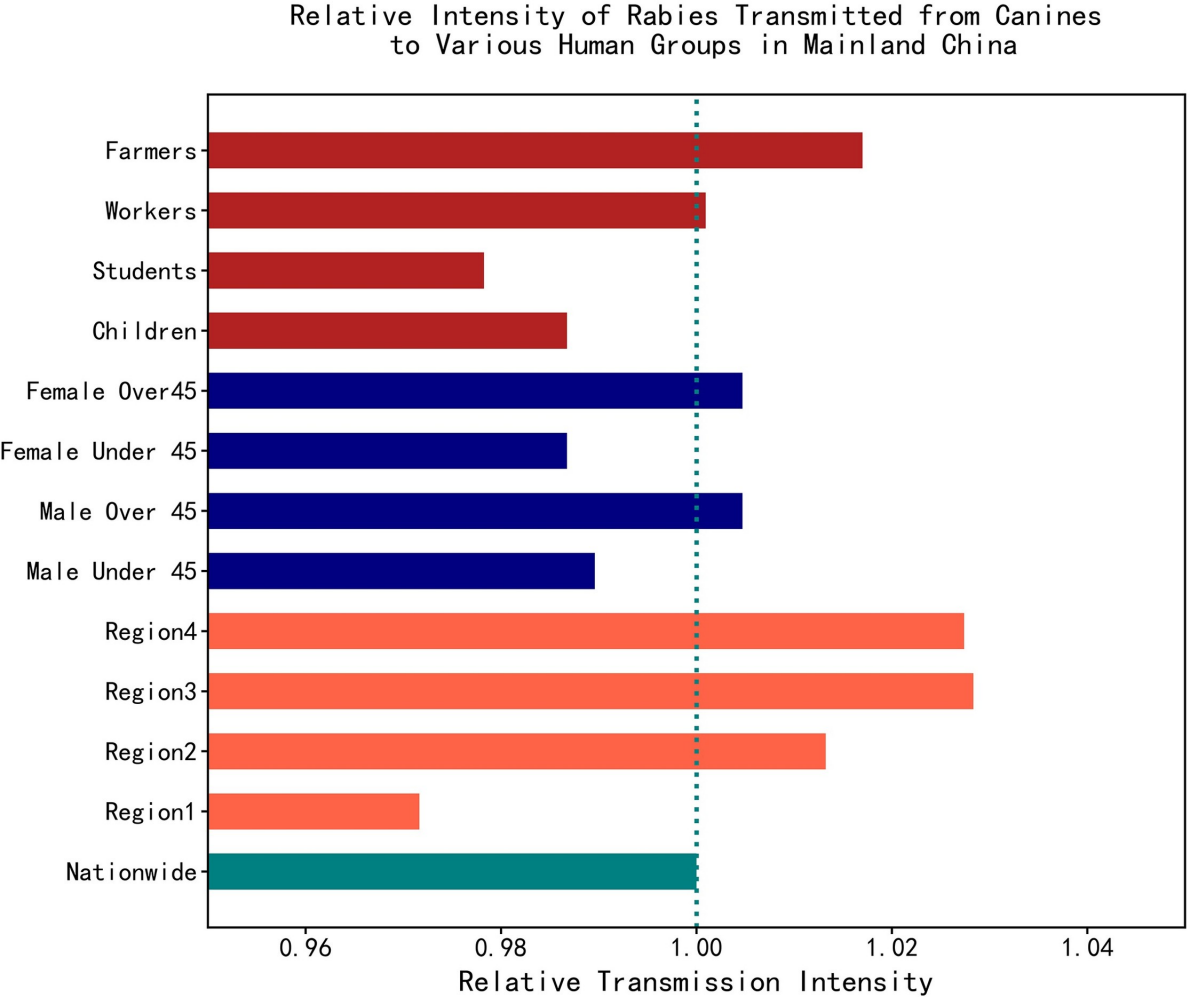
The transmission intensity of rabies from canines to each human population group. The teal color represents the nationwide transmission intensity, whereas the tomato, navy and firebrick color represent transmission intensity of rabies from canines to different regions, age and gender groups and occupational groups, respectively.

The calculated basic reproduction number of canine rabies, R_0_, is marked out in each subgraph in Fig 5, and the relative reproduction numbers for different human groups are summarized in Fig 6. The relative R_0_s of different subgroups were normalized with reference to the R_0_ nationwide, and they can reflect the transmission intensity of rabies from canines to different groups to a certain extent. The calculated R_0_ of canine rabies nationwide is 1.058. We found that the calculated R_0_ of the canine rabies varied in accordance to different population groups. This finding was inconsistent with the results of epidemiological descriptive analysis. The R_0_s calculated on the basis of region I, age groups under 45 years old, and occupation groups including students and children were lower than the nationwide level. This finding indicates that the proportion of human deaths from rabies in these populations decreased during the past decade. Meanwhile, the R_0_s calculated using the other groups were higher than the nationwide level. This finding reflected the increasing proportion of human deaths from rabies and higher transmission intensity from canines to those groups, as shown in Fig 6. All the calculated R_0_s were greater than 1.0, indicating rabies would not gradually disappear in canines. We believe that the diversity of the calculated R_0_s presented the difference in canine and human rabies prevention and control strength and the cognition of rabies for different populations. In mainland China, human rabies was mainly transmitted from canines, and preventing and controlling canine and human rabies were still momentous tasks from the perspective of epidemiological reports and the numerical simulation results.

### Sensitivity analysis

**Fig 7:**
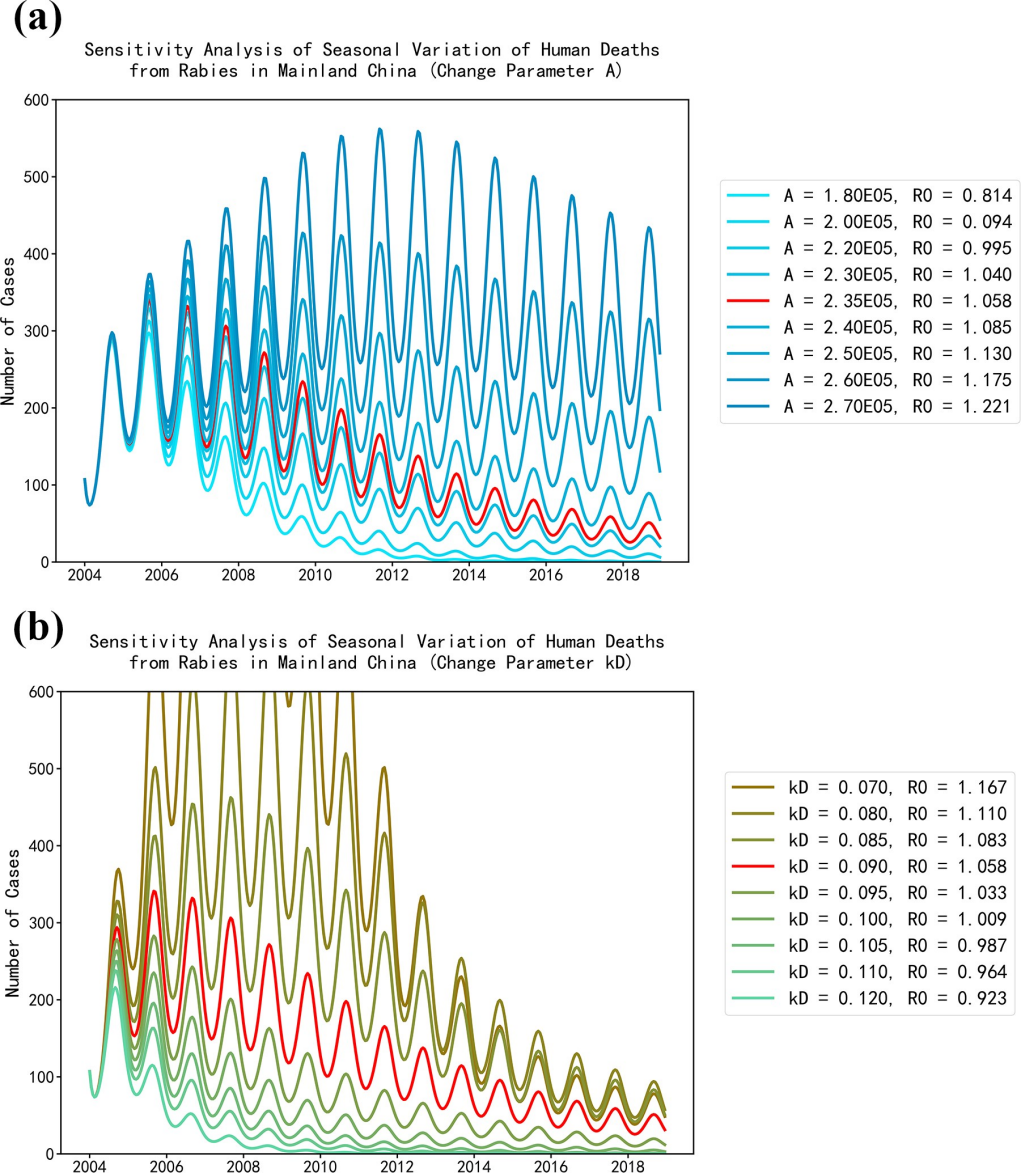
Sensitivity analysis of seasonal variation of human deaths from rabies in mainland China. (a) The impact of birth rate of canines. (b) The impact of vaccination rate of canines.

We explored the impact of changing part of the parameters related to canines on the number of human deaths from rabies, taking the number of nationwide human deaths from rabies as an example. Fig 7A shows the impact of changing the birth rate of canines on human deaths from rabies nationwide. The red curve represents the simulation result using the real monthly data of human deaths from rabies, and the gradual blue curves represent the simulation results of human deaths from rabies by changing the birth rate of canines in the beginning of 2004. The legend illustrates the selected birth rates of canines and calculated basic reproduction numbers. We found the birth rate of canines had an important impact on the number of human deaths from rabies. The decline of the birth rate of canines significantly leads to the decrease of the number of human deaths from rabies. When the birth rate of canines decreased from 2.34×10^5^ month^-1^ to 2.20×10^5^ month^-1^, the basic reproduction number of rabies of canines would be less than 1.0. Therefore, we believe that if effective measures, such as the artificial sterilization of some wild or domestic pet canines, can be taken to reduce the birth rate of canines in the whole country, especially in high risk areas, the spread of rabies can be controlled in canines and the spread of rabies from canines to humans can be effectively prevented.

Fig 7B shows the impact of changing the immunization rate of canines on human deaths from rabies nationwide. The red curve represents the simulation result using the real monthly data of human deaths from rabies, and the gradual green to yellowish brown curves represent the simulation results of human deaths from rabies by changing the immunization rate of canines in the beginning of 2004. The legend illustrates the selected immunization rates of canines and calculated basic reproduction numbers. We found the immunization rate of canines also had an important impact on the number of human deaths from rabies. The increase of the immunization rate of canines significantly leads to the decrease of the number of human deaths from rabies. When the immunization rate of canines increased from 0.90 month^-1^ to 0.105 month^-1^, the basic reproduction number of rabies of canines would be less than 1.0. Therefore, we believe that if the rabies prevention and control departments and the public can pay more attention to the immunization of canines, the spread of rabies in canines and the spread of rabies from canines to humans can be effectively prevented.

## Discussion

Since the launch of the infectious disease surveillance system in 2004, detailed epidemic reports have been provided every year. The annual human deaths from rabies in mainland China reached its peak in 2007 and exhibited a significant seasonal pattern. The local governments have been aware of the urgency of the prevention and control of rabies, and have increased the investment to curb its spread because the death rate was as high as 100%. Especially in high risk areas, stricter measures have been considered by the local governments and disease control departments. To date, the annual human deaths from rabies have dropped to less than 500. However, human rabies remained a significant health problem that requires attention in mainland China, and the severity and the economic burden were still inferior to India. Rabies is a type of zoonosis, which is mostly related to mammals, including bats, raccoons, canines, and cats. Human rabies has almost disappeared in Europe and North America due to long-term effective immunization, especially by spreading baits containing vaccine in the wild[34] to prevent the spread of rabies from wild animals to humans. In China, owing to economic development in the past 20 years, the Chinese government has increased its investment in rabies control, and more than 100 million rabies vaccines were consumed every year[33]. Nevertheless, the vaccines were usually used limited to humans and domestic canines and cats, and reports on large-scale vaccine investment for wild mammalian rabies were few.

We applied a continuous deterministic model to simulate human deaths from rabies in different population groups in mainland China without considering the stochastic effect and social factors including canine migration, and human migration. The models were completely consistent; thus, the results from different human groups could be compared, and could reflect the epidemic and the prevention in various areas. We believe that the division according to regions, age and gender groups, and occupational groups could reflect the different living environments, including economic and health conditions, disease prevalence and prevention of various human groups.

We divided mainland China into five different regions, of which region I, containing four states and territories, was the highest risk area. Region II contains the states and territories bordering region I, and region III represents the states and territories adjacent to regional II, excluding those contained in regional I. Epidemiological data showed that rabies had migrated from the south of China to the north. In region I, the annual proportion of human deaths from rabies has decreased from nearly 60% to less than 40%, and the corresponding R_0_ of canine rabies was lower than the nationwide level. This finding reflected that the governments in areas with higher number of human rabies cases attached the highest attention of rabies prevention and control. Meanwhile, in traditional relatively low risk areas, before 2007, insufficient investment by the local governments may result in the cross regional spread of canine and human rabies through canine trade. Phylogenetic analysis demonstrated that rabies has been transmitted between different provinces in mainland China[35–37]. This finding was consistent with the result showing that the annual human deaths from rabies in region I reached a peak one year ahead of other regions and significant decrease in the proportion of human deaths from rabies.

Human deaths from rabies of different gender, age, and occupational population groups reflected the various living environments including economic activities, education, and health services in different living areas. Epidemiological analysis showed that the ratio of men to women in the number of human rabies was approximately 2.37. This finding might be due to the fact that according to the occupational distribution of human deaths from rabies, the morbidity of farmers accounted for the highest proportion. However, in rural areas, men were more inclined to take part in farm work, making them easily accessible to sick stray canines, whereas women spent more time working at home. Interestingly, the calculated R_0_s of canine rabies exhibited no significant gender difference. For age groups of under 45 years old, the calculated R_0_s were lower than the nationwide level. However, the result was the opposite for age groups of over 45 years old. In terms of various occupational groups, the calculated R_0_ of students was the lowest, followed by children, but it exceeded the nationwide level for the farmers. We believe that the different performance of the calculated R_0_s for different groups reflected the cognition of rabies in different regions, different income populations, and different education groups in mainland China. According to the China Statistical Yearbook[31,38], during the past 20 years, rapid development of the economy in mainland China complemented the rapid urbanization. Considerable young rural labor forces that used to be farmers have migrated into cities and became workers. Meanwhile, the investment in education in China has increased year by year. Thus, higher level education gradually became popular, and college students were more inclined to settle down in cities after graduation. This process has led to the continuous expansion of the students, workers and, service industry groups, the continuous absorption of young rural labor forces by cities, and the rapid contraction of farmers. This finding corresponds to the prominent aging of rural areas given that the migration proportion of rural labor forces over 45 years old to urban labor forces was relatively low. In urban areas, students could access better education, and the white collared individuals usually had higher education background and higher income, leading to their higher awareness of rabies and higher motivation for PEP. The urban medical service facilities were superior to those in rural areas, and seeking PEP services was still inconvenient for farmers. Education, an economic burden of local residents, affected the different R_0_ values of canines. The calculated R_0_ based on age groups over 45 years old was higher than that of the young groups, and that bases on students was the lowest among various occupational groups. Children are increasingly born in urban areas with better living conditions, corresponding to a lower R_0_ than that of the nationwide level. Therefore, the transmission intensity of rabies from canines to people over 45 years old are higher than that to the other age groups.

For different occupational groups in mainland China, the absolute number of farmers declined rapidly, and that of other populations increased. However, the R_0_ of canines calculated based on farmers was the highest, and the mortality proportion of farmers related to rabies increased year by year. In some undeveloped areas, the local government and the disease control and prevention department could not supply adequate funds to provide sufficient animal rabies vaccines as well as better management for stray canines. The news of killing stray canines to eliminate canine and human rabies in rural areas appeared occasionally on the China Internet. According to the previous research, immunization was feasible to eliminate rabies. Thus, strengthening the vaccination of stray mammals in rural areas is an important task in the future.

On the basis of current research, we believe that schools could serve as a centralized source of education and publicity and play a key role in the spread of rabies knowledge. However, if China intends to eliminate human rabies completely in the future, publicity should be enhanced, health services among populations, who are more likely to ignore the rabies including people over 45 years old, farmers, and less educated population, should be improved, and better employment opportunities should be provided to lower income people to reduce the economic burden of PEP.

Human cases are mainly transmitted from mammals given that rabies is a type of zoonosis. The publicity and education of PEP for humans should be strengthened, and the immunization of canines, cats, and other mammals, should be enhanced, especially to strengthen the management of wild canines by feeding bait containing vaccines in high risk areas. Human, animal, and environmental health are considered simultaneously, and communication and cooperation among public health professionals, doctors, and veterinarians are required to eliminate human rabies from the source and achieve the goal of One Health[39].

To effectively control the spread of rabies, effective measures should be taken by the rabies prevention and control departments and the public. Although the annual human deaths from rabies is decreasing year by year, the basic reproduction number of rabies of canines is still greater than 1.0. Therefore, the transmission of rabies in canines has not been fundamentally controlled. According to our analysis, properly reducing the birth rate of canines or improving the immunization rate of canines can reduce the basic reproduction number of rabies in canines to less than 1.0. Therefore, the implementation of the above two measures needs to be further strengthened to effectively prevent the spread of rabies in canines and from canines to humans.

However, the data of canines and birth rate of canines were estimated through the monitoring results of several monitoring sites of China CDC, the data of different population groups were referred to relevant information, such as the statistical yearbooks, and some parameters were referred to the values in previous reports. Meanwhile, the data of human deaths from rabies were outputted from the reporting system of China CDC. Some rabies cases may not be recognized or included in the reporting system, and the reporting effectiveness may vary with regions. Therefore, they may cause errors in the calculated R_0_s, indicating the limitations of our study. In addition, because the monthly human deaths from rabies in China have been constantly decreasing, the human deaths from rabies of some population groups fluctuated wildly, thereby resulting in larger fitting relative error. However, fewer human deaths from rabies in some population groups indicated that human rabies has been effectively controlled in these groups. Last but not least, the monthly human deaths from rabies of different regions, age and genders, and occupations were provided independently, we did not conduct a more detailed study of different occupational groups of a certain age and gender group. In the future, we will continue to track the epidemiological characteristics of human rabies in China, continue to revise the existing model to assist in rabies prevention and control departments to formulate more appropriate prevention and control policies, and strive to completely eliminate human rabies in mainland China before 2030.

## Conclusion

In China, animals and human rabies are still major public health problems. We reviewed the epidemiology of human deaths from rabies in mainland China and simulated the human deaths from rabies of different population groups by applying the SEIR model. We discovered that the transmission intensity of rabies from canines to populations of central China, over 45 years old and farmers were higher than the nationwide level, and these groups had higher risk of human rabies. To eliminate human rabies by 2030, the education and publicity of rabies knowledge among these regions and population groups should be further increased, and the immunization of stray canines in the areas where the rabies was more likely to be neglected should be strengthened. The cooperation of various domains involving humans, animals and the environments to achieve the goal of One Health should be enhanced.

## Supporting information

S1 Table Parameters of SEIR model for human deaths from rabies for different regions, different age and gender groups and different occupational groups in mainland China. (PDF)

S2 Table Mann Kendall trend test of different age groups for men and women. (PDF)
