# Supplementary material for: Epidemiological and numerical simulation of rabies spreading from canines to various human populations in mainland China
Source: PLoS Negl Trop Dis. 2021 Jul 14;15(7):e0009527. doi: 10.1371/journal.pntd.0009527 (PMC8312940; doi:10.1371/journal.pntd.0009527)
Supplement: S1 Table — (PDF) [file pntd.0009527.s001.pdf]

S1_Table

Parameters of SEIR model for human deaths from rabies for different regions, different age and gender groups and different occupational groups in mainland China.

Parameters	Male<45	Male>45	Female<45	Female>45
A	2.34×10^5	2.34×10^5	2.34×10^5	2.34×10^5
λ_D	0.1666	0.1666	0.1666	0.1666
γ_D	0.49	0.49	0.49	0.49
σ_D	0.95	0.95	0.95	0.95
m_D	0.0064	0.0064	0.0064	0.0064
k_D	0.09	0.09	0.09	0.09
μ_D	1	1	1	1
B	7.00×10^5	8.72×10^5	6.40×10^5	8.63×10^5
λ_H	0.1666	0.1666	0.1666	0.1666
γ_H	0.5	0.5	0.5	0.5
σ_H	0.5	0.5	0.5	0.5
m_H	0.001912	0.001783	0.001988	0.00164
k_H	0.54	0.54	0.54	0.54
μ_H	1	1	1	1
S_D	3.30×10^7	3.30×10^7	3.30×10^7	3.30×10^7
E_D	2.20×10^4	2.20×10^4	2.20×10^4	2.20×10^4
I_D	1.10×10^4	1.10×10^4	1.10×10^4	1.10×10^4
R_D	3.30×10^6	3.30×10^6	3.30×10^6	3.30×10^6
S_H	4.56×10^8	2.03×10^8	4.34×10^8	2.03×10^8
E_H	110	66	110	54
I_H	55	33	55	27
R_H	2.10×10^7	9.37×10^6	2.00×10^7	9.37×10^6
a_D	9.85×10^{-8}	1.00×10^{-7}	9.82×10^{-8}	1.00×10^{-7}
a_H	2.84×10^{-11}	4.64×10^{-11}	1.28×10^{-11}	2.38×10^{-11}
b_D	0.42	0.42	0.42	0.42
b_H	0.33	0.33	0.33	0.33

S1_Table continued

Parameters	Region1	Region2	Region3	Region4
A	4.15×10^{4}	5.50×10^{4}	5.25×10^{4}	6.50×10^{4}
λ_D	0.1666	0.1666	0.1666	0.1666
γ_D	0.49	0.49	0.49	0.49
σ_D	0.95	0.95	0.95	0.95
m_D	0.0064	0.0064	0.0064	0.0064
k_D	0.09	0.09	0.09	0.09
μ_D	1	1	1	1
B	2.87×10^{5}	3.11×10^{5}	2.98×10^{5}	3.66×10^{5}
λ_H	0.1666	0.1666	0.1666	0.1666
γ_H	0.5	0.5	0.5	0.5
σ_H	0.5	0.5	0.5	0.5
m_H	0.000632	0.000651	0.000643	0.000666
k_H	0.54	0.54	0.54	0.54
μ_H	1	1	1	1
S_D	6.04×10^{6}	7.88×10^{6}	7.15×10^{6}	9.17×10^{6}
E_D	4.03×10^{3}	5.25×10^{3}	4.77×10^{3}	6.11×10^{3}
I_D	2.01×10^{3}	2.63×10^{3}	2.38×10^{3}	3.06×10^{3}
R_D	6.04×10^{5}	7.88×10^{5}	7.15×10^{5}	9.17×10^{5}
S_H	2.39×10^{8}	3.10×10^{8}	2.82×10^{8}	3.61×10^{8}
E_H	170	34	44	38
I_H	85	17	22	19
R_H	1.10×10^{7}	1.10×10^{7}	1.43×10^{7}	1.30×10^{7}
a_D	5.45×10^{-7}	4.29×10^{-7}	4.56×10^{-7}	3.68×10^{-7}
a_H	4.28×10^{-10}	6.53×10^{-11}	5.58×10^{-11}	2.88×10^{-11}
b_D	0.42	0.42	0.42	0.42
b_H	0.23	0.43	0.43	0.43

S1_Table continued

Parameters	Children	Students	Workers	Farmers
A	2.34×10^{5}	2.34×10^{5}	2.34×10^{5}	2.34×10^{5}
λ_D	0.1666	0.1666	0.1666	0.1666
γ_D	0.49	0.49	0.49	0.49
σ_D	0.95	0.95	0.95	0.95
m_D	0.0064	0.0064	0.0064	0.0064
k_D	0.09	0.09	0.09	0.09
μ_D	1	1	1	1
B	1.34×10^{6}	1.67×10^{6}	2.57×10^{5}	6.70×10^{5}
λ_H	0.1666	0.1666	0.1666	0.1666
γ_H	0.5	0.5	0.5	0.5
σ_H	0.5	0.5	0.5	0.5
m_H	0.0119	0.00694	0.002	0.0006
k_H	0.54	0.54	0.54	0.54
μ_H	1	1	1	1
S_D	3.30×10^{7}	3.30×10^{7}	3.30×10^{7}	3.30×10^{7}
E_D	2.20×10^{4}	2.20×10^{4}	2.20×10^{4}	2.20×10^{4}
I_D	1.10×10^{4}	1.10×10^{4}	1.10×10^{4}	1.10×10^{4}
R_D	3.30×10^{6}	3.30×10^{6}	3.30×10^{6}	3.30×10^{6}
S_H	1.12×10^{8}	2.40×10^{8}	2.00×10^{8}	6.50×10^{8}
E_H	34	46	18	174
I_H	17	23	9	87
R_H	5.17×10^{6}	1.11×10^{7}	9.23×10^{6}	3.00×10^{7}
a_D	9.82×10^{-8}	9.74×10^{-8}	9.96×10^{-8}	1.01×10^{-7}
a_H	3.28×10^{-11}	2.90×10^{-11}	1.26×10^{-11}	2.52×10^{-11}
b_D	0.42	0.42	0.42	0.42
b_H	0.33	0.33	0.33	0.33
