# Supplementary material for: Epidemiological and numerical simulation of rabies spreading from canines to various human populations in mainland China
Source: PLoS Negl Trop Dis. 2021 Jul 14;15(7):e0009527. doi: 10.1371/journal.pntd.0009527 (PMC8312940; doi:10.1371/journal.pntd.0009527)
Supplement: S2 Table — (PDF) [file pntd.0009527.s002.pdf]

S2_Table

Mann Kendall trend test of different age groups for men and women.

Male Age Group	Proportion	95CI	mk_test_Trend	h	p	z
0-5	0.0306	(0.0231,0.0382)	decreasing	TRUE	0.029448	-2.1774
5-10	0.0467	(0.0190,0.0744)	decreasing	TRUE	0.000007	-4.5089
10-15	0.0397	(0.0080,0.0713)	decreasing	TRUE	0.000001	-4.8497
15-20	0.0186	(-0.0011,0.0383)	decreasing	TRUE	0.000005	-4.5528
20-25	0.0162	(0.0070,0.0254)	no trend	FALSE	0.074825	-1.7815
25-30	0.0201	(0.0137,0.0266)	no trend	FALSE	0.276278	-1.0887
30-35	0.0273	(0.0165,0.0381)	decreasing	TRUE	0.047761	-1.9795
35-40	0.0377	(0.0224,0.0529)	decreasing	TRUE	0.029448	-2.1774
40-45	0.0572	(0.0365,0.0779)	no trend	FALSE	0.42848	-0.7918
45-50	0.0612	(0.0325,0.0900)	increasing	TRUE	0.007533	2.6723
50-55	0.0685	(0.0455,0.0915)	no trend	FALSE	0.766525	0.2969
55-60	0.0737	(0.0419,0.1055)	no trend	FALSE	0.373053	0.8908
60-65	0.0729	(0.0324,0.1133)	increasing	TRUE	0.000005	4.5528
65-70	0.0563	(0.0193,0.0934)	increasing	TRUE	0.000169	3.761
70-75	0.0391	(0.0259,0.0523)	increasing	TRUE	0.000765	3.3651
75-80	0.0229	(0.0056,0.0402)	increasing	TRUE	0.004101	2.8703
80-85	0.0091	(0.0022,0.0159)	increasing	TRUE	0.007533	2.6723
85-100	0.0032	(-0.0005,0.0069)	increasing	TRUE	0.000113	3.86

S2_Table continued

Female Age Group	Proportion	95CI	mk_test_Trend	h	p	z
0-5	0.0163	(0.0078,0.0248)	decreasing	TRUE	0.029448	-2.1774
5-10	0.0237	(0.0083,0.0390)	decreasing	TRUE	0.029448	-2.1774
10-15	0.0173	(0.0069,0.0277)	decreasing	TRUE	0.000113	-3.86
15-20	0.0066	(0.0010,0.0123)	decreasing	TRUE	0.000169	-3.761
20-25	0.0038	(-0.0004,0.0081)	decreasing	TRUE	0.022822	-2.2764
25-30	0.0055	(0.0004,0.0106)	no trend	FALSE	0.921159	-0.099
30-35	0.0079	(0.0011,0.0147)	decreasing	TRUE	0.001539	-3.1672
35-40	0.0142	(0.0035,0.0250)	decreasing	TRUE	0.000765	-3.3651
40-45	0.02	(0.0111,0.0288)	decreasing	TRUE	0.002153	-3.0682
45-50	0.022	(0.0088,0.0352)	no trend	FALSE	0.692181	-0.3959
50-55	0.0297	(0.0165,0.0428)	no trend	FALSE	0.3223	-0.9897
55-60	0.0303	(0.0163,0.0444)	no trend	FALSE	0.843085	-0.1979
60-65	0.0311	(0.0166,0.0456)	increasing	TRUE	0.000075	3.959
65-70	0.0256	(0.0043,0.0469)	increasing	TRUE	0.017531	2.3754
70-75	0.0222	(0.0104,0.0339)	increasing	TRUE	0.013348	2.4744
75-80	0.0134	(0.0066,0.0201)	no trend	FALSE	0.3223	0.9897
80-85	0.0065	(0.0002,0.0127)	increasing	TRUE	0.047761	1.9795
85-100	0.0029	(-0.0012,0.0070)	no trend	FALSE	0.399612	0.8423
